# A Novel Calcium-Dependent Protein Kinase 1 Inhibitor Potently Prevents *Toxoplasma gondii* Transmission to Foetuses in Mouse

**DOI:** 10.3390/molecules26144203

**Published:** 2021-07-10

**Authors:** Héloïse Débare, Nathalie Moiré, Firmin Baron, Louis Lantier, Bruno Héraut, Nathalie Van Langendonck, Caroline Denevault-Sabourin, Isabelle Dimier-Poisson, Françoise Debierre-Grockiego

**Affiliations:** 1 Infectiologie et Santé Publique, National Research Institute for Agriculture, Food and the Environment, Université de Tours, 37000 Tours, France; heloise.debare@gmail.com (H.D.); nathalie.moire@univ-tours.fr (N.M.); firmin.baronc2i@gmail.com (F.B.); louis.lantier@univ-tours.fr (L.L.); bruno.heraut@univ-tours.fr (B.H.); dimier@univ-tours.fr (I.D.-P.); 2 Service de Parasitologie-Mycologie-Médecine Tropicale, Centre Hospitalier Régional Universitaire de Tours, 37000 Tours, France; langendo@univ-tours.fr; 3 Groupe Innovation et Ciblage Cellulaire EA7501, Université de Tours, 37200 Tours, France; caroline.denevault@univ-tours.fr

**Keywords:** *Toxoplasma gondii*, congenital toxoplasmosis, treatment, imidazoazines

## Abstract

Treatments currently used to prevent congenital toxoplasmosis are non-specific of *Toxoplasma gondii* and have grievous side effects. To develop a more specific and less toxic drug, we have designed SP230, an imidazo[1,2-*b*]pyridazine salt targeting the *Toxoplasma gondii* calcium-dependent protein kinase 1 (*Tg*CDPK1) and active against acute toxoplasmosis in mice. Efficiency of SP230 to inhibit foetal transmission of the parasite was evaluated in a mouse model of congenital toxoplasmosis. Swiss mice were infected at mid-pregnancy with tachyzoites or cysts of the ME49 strain of *T. gondii* by intraperitoneal and oral route, respectively, and treated with SP230 at 50 mg/kg for 5 days by the same routes. Parasite burden in organs of dams and in foetuses was measured by quantitative PCR. Intraperitoneal administration of SP230 drastically reduced the number of parasites (more than 97% of reduction) in the brain and lungs of dams, and led to a reduction of 66% of parasite burden in foetuses. Oral administration of SP230 was particularly efficient with 97% of reduction of parasite burdens in foetuses. SP230 did not impact number and weight of offspring in our conditions. This inhibitor of *Tg*CDPK1 is a promising candidate for the development of alternative therapeutics to treat infected pregnant women.

## 1. Introduction

Human beings are at risk of infection with the apicomplexan parasite *Toxoplasma gondii* mainly by ingestion of cysts present in undercooked meat. After ingestion, bradyzoites are liberated from cysts and differentiate into tachyzoites, the parasite form replicating within the host cells. In pregnant women, tachyzoites can cross the placental barrier and infect the foetus resulting in congenital toxoplasmosis. The probability of foetal infection and the severity of further complications depend on the gestational age at the time of infection. During the first trimester, the trans-placental passage is less likely, but in case of foetus contamination, the damages are often severe, characterized by the classical triad of chorioretinitis, intracranial calcifications or hydrocephalus [1]. As pregnancy progresses, the rate of vertical transmission proportionally increases, resulting in the second trimester, in preterm births and/or neurologic and ocular disorders in new-born babies. Finally, during the third trimester, the foetal infection is of high risk but generally subclinical, with latent forms, sometimes leading to delayed neurological or ophthalmological sequelae [2]. The macrolide antibiotic spiramycin is often used to inhibit transmission of infection to the foetus but is inefficient when foetal contamination is confirmed, because it cannot cross the placental barrier [3]. At this stage, the therapeutic option widely used is an association of pyrimethamine with a sulphonamide drug, targeting the *T. gondii* folate pathway, characterized by a modest efficacy, long periods of treatment and significant side effects [4,5]. Novel treatment options are thus required.

A large number of anti-*Toxoplasma* compounds has been described in the literature, but very few studies on drug efficacy in a congenital model are available [6]. We have previously demonstrated the outstanding efficacy of the imidazo[1,2-*b*]pyridazine salt SP230, a *T. gondii* calcium-dependent protein kinase 1 (*Tg*CDPK1) inhibitor that strongly reduced parasite burdens in the brain and lungs of non-pregnant infected mice [7]. In addition, we have shown that SP230 exhibited excellent systemic exposure, with a high blood–brain barrier penetration in mice when administered by intraperitoneal route [7].

We here report on the safety and efficacy of SP230 against vertical transmission of *T. gondii*. Like humans, mice are natural intermediate hosts of *T. gondii* and because of the high number of offspring per litter, robust statistical analyses are possible with only few pregnant Swiss females. Mice were infected at mid-pregnancy, the optimal time for foetal transmission without abortion. In our previous study, SP230 was administered by intraperitoneal route to mice infected by the same route. Since natural infection and treatment of pregnant women occur by oral route, we here compared both routes of infection/treatment.

## 2. Results

### 2.1. Chemical Synthesis of SP230 and Validation of Its Therapeutic Index

The batch of SP230 synthesized for this study had similar therapeutic index than in our previous work [7], with a CE_50_ of 0.080 µM on *T. gondii* tachyzoites in vitro and a CC_50_ of more than 30 µM on the human foreskin fibroblasts used as host cells for tachyzoite growth in vitro (CC_50_/CE_50_ > 350).

### 2.2. Establishment of Specific Standard Curves for qPCR

To evaluate the efficiency of *T. gondii* inhibitors, the qPCR method is often used to detect the parasites in organs of treated animals and a standard curve obtained with known amount of tachyzoites is used to precisely quantify them [8,9]. Since *T. gondii* DNA is extracted together with organ DNA, we wanted to decipher whether this host DNA interferes with the amplification of the parasite DNA. For this, we performed four different standard curves with known amounts of tachyzoites alone or added to brain, lungs or foetus from non-infected mice. By this way, we found out that the crossing point (minimum number of cycles required to detect amplified DNA) was different according to the type of tissue (i.e., of crossing points for 1000 tachyzoites: 23.6 in brain, 21.2 in lungs, 23.8 in foetus and 22.9 for tachyzoites alone). Processing of DNA of non-infected tissue gave no crossing point value. In light of these results, parasite burdens in infected mice were calculated with the appropriate standard curve.

### 2.3. SP230-Treatment of Pregnant Mice Infected with T. gondii

**Figure 1:**
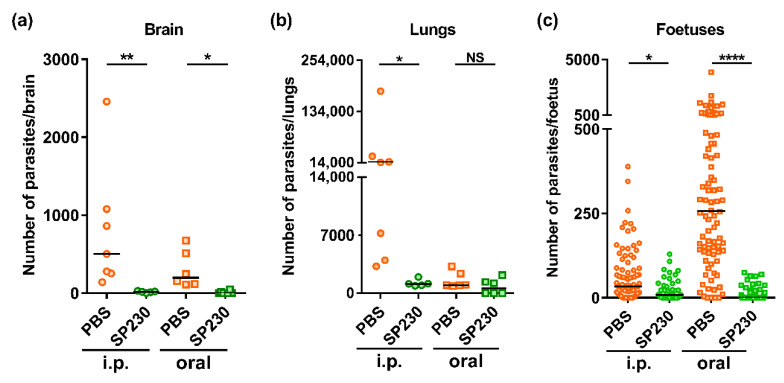
Mice were infected at mid-gestation by tachyzoites (intraperitoneal route, i.p.) or cysts (oral route) of the ME49 strain of *T. gondii*. Mice received PBS or SP230 (50 mg/kg) by the same routes for 5 days. The number of *T. gondii* parasites was evaluated by qPCR at day 17 of gestation in brain (**a**) and lungs (**b**) of dams (*n* = 5 to 7) or in foetuses ((**c**), *n* = 90 [PBS i.p., 5 dams], 47 [SP230 i.p., 3 dams], 86 [PBS oral, 6 dams] and 53 [SP230 oral, 3 dams]). NS: not significant, * *p* < 0.05, ** *p* < 0.01, **** *p* < 0.0001 (Dunn’s multiple comparison test).

To evaluate the efficiency of SP230 to inhibit the transmission of *T. gondii* to foetus, mice were infected at mid-gestation either with tachyzoites by intraperitoneal route or orally with cysts and treated with SP230 administered by the same route than infection. After 17 days of gestation, parasite burden was quantified by qPCR in brain and lungs of dams and in foetuses. As shown in Figure 1a, both intraperitoneal and oral treatments of pregnant mice with SP230 significantly reduced parasite burdens in brain of dams with global reduction of 98% (*p* < 0.01) and 96% (*p* < 0.05), respectively. The decrease in parasite numbers observed in lungs of dams was significant (*p* < 0.05) after intraperitoneal treatment (97%), but not after oral treatment (49%). This might be explained by the 23-fold lower number of parasites in the lungs of untreated mice infected orally compared to those infected by the intraperitoneal route (Figure 1b). In contrast to these results on dams, the inhibition of vertical transmission of *T. gondii* was more efficient after oral treatment (Figure 1c). Indeed, the reduction in parasite burdens in foetuses reached 97% when dams were orally treated with SP230 (*p* < 0.0001), while the diminution was of 66% in the case of intraperitoneal administration (*p* < 0.05). Contrary to the dams, the number of parasites in foetuses was more elevated when mice were infected orally, and the significance of the difference with the treated group was consequently more stringent.

### 2.4. SP230-Treatment of Pregnant Non-Infected Mice

In the experiment described in the (Section 2.3), the number of foetuses was not significantly different after SP230 treatment of *T. gondii*-infected mice compared to the control groups (PBS i.p.: 18 ± 3 and SP230 i.p.: 16 ± 2, *p* value = 0.439; PBS p.o.: 14 ± 1 and SP230 p.o.: 18 ± 1, *p* value = 0.0838, Dunn’s multiple comparisons test after Kruskal-Wallis test). To firmly exclude a toxic effect of SP230 during pregnancy, non-infected mice were treated following the same schedule as for the previous experiment. The number of offspring was slightly higher in the group of animals that received PBS per oral route, but the difference with the SP230 group was not significant (Table 1). The number of offspring was very similar when mice received PBS or SP230 by the intraperitoneal route. At 4 weeks, the mean weight of female and male offspring born from mice treated with SP230 by the intraperitoneal route was higher than those of female and male offspring born from control mice (PBS i.p.). No significant difference in weight was observed between the two groups from animals orally treated (Table 1).

In conclusion, oral administration of imidazoazine SP230 provided strong protection against foetal infection by *T. gondii* in mouse without negative effect on the progeny in terms of offspring number and growth in the present therapeutic protocol.

## 3. Discussion

Toxoplasmosis is the first parasitic foodborne disease responsible for high healthcare costs. For few years, guidelines for prevention, diagnosis and treatment of congenital toxoplasmosis have been published by working groups from different countries to prevent complications [3,10,11,12,13]. In France and Austria, systematic serological screening of anti-*Toxoplasma* antibodies in pregnant women permits for starting the treatment very early after infection. On the contrary, the cost of diagnosis is estimated to be too high in the USA to be generalized to all pregnant women, leading to an underestimation of the number of cases [11,14,15]. As a consequence of these different public health policies, the rate of severe clinical signs like hydrocephaly is much higher in the USA than in France in infants congenitally infected with the same type of *T. gondii* strain [16,17,18]. A retrospective analysis of 11 studies has estimated the rate of vertical transmission at 9.9% after therapy [4], indicating that it is not optimal. This could be due to inefficacy of the first line treatments on sulfadiazine-resistant strains [19]. Furthermore, hematologic disorders like neutropenia (1.7 to 45.8%), anaemia (0.8 to 50%), thrombocytopenia (6.9 to 7.7%) and eosinophilia (26.2%) are often observed in patients with maternal and congenital toxoplasmosis treated with pyrimethamine [20], highlighting the importance of developing alternative therapeutics more specific without undesirable side effects.

Among *Tg*CDPK1 inhibitors, the bumped kinase inhibitor BKI-1294 demonstrated excellent efficiency against trans-placental transmission of *T. gondii* in pregnant mice (reduction > 90%) but with potential detrimental effect on fertility [8]. When BKI-1294 was administered to ewes from mid-gestation, no abortion and no loss of weight was observed at birth of lambs [21]. In our present study, there were 12 to 19 and 14 to 19 offspring per litter in control (PBS) and SP230-treated groups, respectively, suggesting that our *T. gondii* inhibitor does not affect mouse gestation at the tested dose. This was confirmed by the treatment of non-infected mice. In addition, administration of SP230 for 8 days did not trigger significant toxicity in liver and kidney of mice [7], but its administration to other animal species will be nonetheless necessary to conclude to its innocuousness during pregnancy before any use in human. It is noteworthy that SP230, administered by the intra-peritoneal route, drastically reduced the parasite load in brain and lungs as efficiently in pregnant mice (the present study) as in non-pregnant mice [7], suggesting that gestation does not impact the bio-distribution of SP230.

When ewes were infected with *T. gondii* at mid-gestation, the treatment with BKI-1294 increased survival of lambs (from 0 without treatment to 76%), but DNA of *T. gondii* was detected in the brain of 46% of the lambs born alive [21]. BKI-1294 is thus not totally efficient in inhibiting vertical transmission of *T. gondii* in ewes. In our conditions, parasite DNA was not detected in all foetuses either in treated or untreated mice infected with *T. gondii* tachyzoites or cysts. The percentages of foetuses negative for infection increased from 21% and 5% (for mice that have received PBS by i.p. route and oral route, respectively) to 40% and 62% (for mice that have received SP230 by i.p. route and oral route, respectively). In addition, *T. gondii*-positive foetuses from SP230-treated mice presented lower parasite loads than *T. gondii*-positive foetuses of the untreated groups.

Although the protective effect of SP230 against congenital toxoplasmosis should be confirmed on offspring (significant increased survival, decreased ocular damages and parasite load), these results are encouraging for the development of a new treatment of congenital toxoplasmosis.

## 4. Materials and Methods

### 4.1. Chemical Synthesis of SP230

The compound 2-(benzo[*d*][1,3]dioxol-4-yl)-6-(4-methylpiperazin-1-yl)-3-(pyridin-4-yl)imidazo[1,2-*b*]pyridazine bis hydrochloride salt (SP230) was synthesized by McSAF (Tours, France) according to Moine et al. [7].

### 4.2. Functional Characterization of SP230

Inhibitory activity of SP230 on the invasion/proliferation of *T. gondii* tachyzoites of the *T. gondii* RH-β-gal strain and absence of cytotoxicity on human foreskin fibroblasts were verified in vitro as previously described [7].

### 4.3. Treatment of T. gondii-Infected Mice with SP230

The number of animals required to have significant differences in the parasite loads was calculated with tools of Anastats.fr (AnaStats Scop ARL, Rilly-sur-Vienne, France). Twenty-four female Swiss mice of 20–30 g were purchased from Janvier Labs (Le Genest-Saint-Isle, France) and maintained in the animal house of the University of Tours at 3 per cage and supplied with water and food ad libitum. The experimental protocol, carried out in accordance with the European Union Directive (2010/63/EU), was approved by the Ethics Committee n°019 and the French Ministry for Research (permit number APAFIS#6622-2016090215037378 v5). After mating, pregnancy was estimated by gain of weight. Between day 8 and 10 of gestation, 2 groups of mice (*n* = 7 for the group 1 and *n* = 5 for the group 2) received by intraperitoneal route 1000 tachyzoites of the *T. gondii* ME49 type 2 strain (*Toxoplasma* Biological Resource Centre, Reims, France) and 2 groups of mice (*n* = 6 for groups 3 and 4) received by oral route 10 cysts of the ME49 strain obtained from the brain of one CBA/J (Janvier Labs) chronically infected mouse (permit number 01915.03). Groups 1 and 3 received 200 µL of PBS for 5 days by intraperitoneal route and oral route, respectively. Groups 2 and 4 received 200 µL of SP230 at 50 mg/kg for 5 days by intraperitoneal route and oral route, respectively. Mice were euthanized by cervical dislocation at day 17 of gestation. Foetuses were immediately delivered by transabdominal incision and euthanized by decapitation.

### 4.4. Quantitation of Parasite Loads by qPCR

DNA of brain and lungs from dams and DNA of foetuses were extracted as described [7]. Quantitative PCR (qPCR) was performed on 250 ng of genomic DNA in a total volume of 20 µL containing LightCycler^®^ Taqman^®^ Master mix (Roche Diagnostics, Meylan, France), 0.5 µM of the 2 primers TG III: 5′-CCT TGG CCG ATA GGT CTA GG-3′; TG IIb: 5′-GGC ATT CCT CGT TGA AGA TT-3′, and 180 nM of the probe 5′-FAM-TGC AAT AAT CTA TCC CCA TCA CGA TGC ATA CTC AC-TAMRA-3′ (Eurofins Genomics, Ebersberg, Germany). The qPCR program was 2 min at 50 °C, 5 min at 95 °C, 50 cycles of 20 s at 95 °C/60 s at 65 °C with the LightCycler^®^ 2.0 Instrument (Roche Diagnostics). Standard curves were generated by extracting DNA of known amounts of tachyzoites alone or with the DNA of brain, lungs or foetus of non-infected mice. The non-parametric Kruskal–Wallis followed by the Dunn’s multiple comparison post-test was used for statistical evaluation (GraphPad Prism 7 software, San Diego, CA, USA).

### 4.5. Survival of Offspring Born from Mice Treated with SP230

Twenty-six female CBA/J of 20–30 g were purchased from Janvier Labs (France) and maintained in the animal house of the University of Tours at 3 per cage and supplied with water and food ad libitum. The experimental protocol, carried out in accordance with the European Union Directive (2010/63/EU), was approved by the Ethics Committee n°019 and the French Ministry for Research (permit number APAFIS#28673-2020121513492800 v4). Between day 8 and 10 of gestation, 2 groups of 9 mice received 200 µL of PBS for 5 days by intraperitoneal route and oral route, respectively and 2 groups of 9 mice received 200 µL of SP230 at 50 mg/kg for 5 days by intraperitoneal route and oral route, respectively. Survival and weight were recorded at 4 weeks of age.

## Figures and Tables

**Table 1:** Number and weight of offspring. CBA/J mice received PBS or 50 mg/kg of SP230 by intraperitoneal (i.p.) or oral route for 5 days during gestation. Weight of offspring was recorded at the age of 4 weeks. Results are expressed as mean ± standard deviation. Statistical differences were calculated with the Dunn’s multiple comparison test between the PBS i.p. and SP230 i.p. groups and between the PBS oral and SP230 oral groups (^ns^ not significant; ^a^ *p* value = 0.0437; ^b^ *p* value = 0.0236).

Treatment of Dams	Number of Offspring	Weight of Female	Weight of Male
PBS i.p. (*n* = 9)	4 ± 2	17.4 ± 1.6	19.6 ± 1.2
SP230 i.p. (*n* = 9)	3.89 ± 1 ^ns^	18.6 ± 2.3 ^a^	21.6 ± 2.4 ^b^
PBS oral (*n* = 9)	5.22 ± 2	17.4 ± 2.4	20.5 ± 1.5
SP230 oral (*n* = 9)	4.11 ± 2 ^ns^	16.7 ± 2.8 ^ns^	21.9 ± 2.3 ^ns^

## Data Availability

The data presented in this study are available on request from the corresponding author.

## Sample Availability

Samples of the congenital experiment (DNA) are available from the authors.
